# Simultaneous determination of rhamnose, xylitol, arabitol, fructose, glucose, inositol, sucrose, maltose in jujube (*Zizyphus jujube* Mill.) extract: comparison of HPLC–ELSD, LC–ESI–MS/MS and GC–MS

**DOI:** 10.1186/s13065-016-0171-2

**Published:** 2016-04-30

**Authors:** Shihao Sun, Hui Wang, Jianping Xie, Yue Su

**Affiliations:** Center for Chinese Medicine Therapy and Systems Biology, Shanghai University of Traditional Chinese Medicine, Shanghai, 201203 China; Key Laboratory in Flavor & Fragrance Basic Research, Zhengzhou Tobacco Research Institute, China National Tobacco Corporation, Zhengzhou, 450001 China

**Keywords:** Carbohydrates, Jujube extract, LC-ELSD, LC-ESI-MS/MS, GC–MS

## Abstract

**Background:**

Jujube extract is commonly used as a food additive and flavoring. The sensory properties of the extract, especially sweetness, are a critical factor determining the product quality and therefore affecting consumer acceptability. Small molecular carbohydrates make major contribution to the sweetness of the jujube extract, and their types and contents in the extract have direct influence on quality of the product. So, an appropriate qualitative and quantitative method for determination of the carbohydrates is vitally important for quality control of the product.

**Results:**

High performance liquid chromatography-evaporative light scattering detection (HPLC-ELSD), liquid chromatography-electronic spay ionization tandem mass spectrometry (LC-ESI-MS/MS), and gas chromatography–mass spectrometry (GC–MS) methods have been developed and applied to determining small molecular carbohydrates in jujube extract, respectively. Eight sugars and alditols were identified from the extract, including rhamnose, xylitol, arabitol, fructose, glucose, inositol, sucrose, and maltose. Comparisons were carried out to investigate the performance of the methods. Although the methods have been found to perform satisfactorily, only three sugars (fructose, glucose and inositol) could be detected by all these methods. Meanwhile, a similar quantitative result for the three sugars can be obtained by the methods.

**Conclusions:**

Eight sugars and alditols in the jujube extract were determined by HPLC-ELSD, LC-ESI-MS/MS and GC–MS, respectively. The LC-ELSD method and the LC-ESI-MS/MS method with good precision and accuracy were suitable for quantitative analysis of carbohydrates in jujube extract; although the performance of the GC–MS method for quantitative analysis was inferior to the other methods, it has a wider scope in qualitative analysis. A multi-analysis technique should be adopted in order to obtain complete constituents of about the carbohydrates in jujube extract, and the methods should be employed according to the purpose of analysis.

**Electronic supplementary material:**

The online version of this article (doi:10.1186/s13065-016-0171-2) contains supplementary material, which is available to authorized users.

## Background

Jujube (*Zizyphus jujube* Mill.) is widely distributed in subtropical areas of the northern hemisphere, especially in China [1]. It has been commonly used as functional foodstuff and crude drug in traditional Chinese medicine [2, 3]. Naturally, jujube extract, extracted from jujube fruit by ethanol, is commonly used as food additive and flavoring and it is also listed in the “Lists of food additive” in China [4].

The sensory properties of jujube extract, especially sweetness, are a critical factor determining the product quality and therefore affecting acceptability of consumers. And the carbohydrates with low molecular weight make major contribution to the sweetness of jujube extract. The existence of those compounds could reduce offensive odor, making the flavor good. Therefore, an appropriate qualitative and quantitative method for small molecular carbohydrates is vitally important for quality control of the jujube extract product.

Due to its stable performance in quantitative analysis, Liquid chromatography coupled to various detectors was the most popular analytical method for determination of small molecular carbohydrates [5–13]. However, chemical structure information of analytes can’t be obtained by the methods, which greatly restricted its application for qualitative analysis. Nowadays, the emergence of mass spectrometry has increased the sensitivity of sample detection by the selection of appropriate molecular and fragment ions to avoid interferences from co-extracted sample materials [14]. With high sensitivity, selectivity and robustness, gas chromatography–mass spectrometry (GC–MS) and liquid chromatography-electrospray ionization tandem mass spectrometry (LC-ESI-MS/MS) have widely applied to all kinds of analytical research to obtain the qualitative and quantitative information of analytes [15]. As a result, mass spectrometry was also employed in combination with chromatography for the analysis of sugars [16].

Generally, the low volatility and poor ionization efficiency of carbohydrates make the step of derivatization indispensable for GC–MS and LC-ESI-MS/MS to achieve a satisfactory analysis. Although LC/MS method using atmospheric pressure chemical ionization (APCI) as ion sourse did not require the derivatization step, CHCl_3_ and CH_2_Cl_2_ were often needed in the pre- or post-column stage to attain a satisfactory sensitivity [17–20]. And better sensitivity could always be obtained by methods using derivatization, with a minimum detectability of several to tens of pg [9]. So, it was unusual now for the LC-APCI-MS method to be employed for qualitative and quantitative analysis of carbohydrates, especially for small molecular sugars.

When GC–MS or LC-ESI-MS/MS was employed for the analysis of carbohydrates, silylation, acetylation, methylation and trifluoroacetylation were the most popular derivatizing techniques [21, 22], but these single-step reactions were not suitable for the analysis of reducing sugar due to the variety of isomers that co-exist in aqueous solution [23, 24]. Therefore, some attempts have been made to reduce the number of chromatographic peaks of each derivatized sugar [21], in which the oximation reaction was found to be effective, since it could convert the cyclic hemiacetals into the corresponding open-chain aldose derivatives [22].

Currently, HPLC-ELSD, GC–MS and LC-ESI-MS/MS have been reported in the separation and determination of sugars. However, it was rare to see the comparison of different methods to measure small molecular carbohydrates in jujube extract. In the study, HPLC-ELSD, LC-ESI-MS/MS and GC–MS methods were respectively developed and applied to analyzing small molecular carbohydrates in jujube extract and the performances of these methods were compared.

## Experimental

### Materials and reagents

Jujube extract, named as J1 was purchased from Zhengzhou Jieshi chemical company, China. It was produced by the following procedure: jujube fruit (*Zizyphus jujube* Mill.) was cleaned of soil and grass and denucleated. The pitted jujubes were then crumbed and heated to reflux in edible alcohol (95 %) which was used as extract solvent. Finally, the jujube extract was obtained after evaporation of the alcohol. As a comparison, a home-made jujube extract (J2) was also prepared in our laboratory by an identical method.

Bond Elut C18 Solid phase extraction (SPE) cartridges (500 mg/6 mL), Bond NH2 SPE cartridges (500 mg/3 mL), Poly-Sery HLB SPE cartridges (60 mg/3 mL) and Bond Carbon-GCB SPE cartridges (250 mg/3 mL) were purchased from CNW, (Shanghai, China).

Rhamnose, xylitol, arabitol, fructose, glucose, inositol, sucrose, maltose and xylose used as internal standard were purchased from Sigma-Aldrich (Shanghai, China). Derivatization reagents including N-methyl-N-(trimethylsilyl) trifluoroacetamide (MSTFA) and methoxyamine hydrochloride, and pyridine used as a solvent were purchased from J&K (Beijing, China). Acetonitrile was HPLC grade (Burdich & Jcakson, Muskegon, MI, USA). HPLC-grade ammonium formate was purchased from Tedia (USA). Unionized Water was obtained from a Milli-Q purification system (Millipore, USA). All the standards and reagents used were of purity higher than 98 % and further unpurified in the paper.

### Sample preparation

#### Sample preparation for LC-ELSD method

100 mg of jujube extract was dissolved in 20 mL of unionized water and ultrosounded for 30 min at ambient temperature, and then 10 mL of the mixture was centrifuged for 10 min by KH-500DE ultrasound apparatus (Kunshan Ultrasound Apparatus Lit. Co., China) at 6000 r/min. 1 mL of the supernatant was deposited in a SPE column pre-eluted by 5 mL of methanol and 5 mL of unionized water in turn, and then the SPE column was eluted by unionized water. The eluate was collected and diluted to a 2.5 mL volumetric flask by unionized water, which was used as the sample for LC-ELSD analysis.

#### Sample preparation for LC–MS/MS method and GC–MS method

25 mg of jujube extract was diluted to a 25 ml volumetric flask by water. After filtered through 0.45 μm micropore film, 10 μL of sample was transferred to a chromatographic bottle and 3 μL of xylose (0.1 mg/mL) as internal standard was added. Subsequently, the solution was dried by an N-EVAP concentrator (Organomation Associates, Inc., Berlin, MA, USA) and the residue was used for the further derivatization.

The derivatization method was mainly based on the published literatures [25–27] and the procedure was as follows: the sample of small molecular carbohydrates was mixed with 50 μL solution of methoxyamine hydrochloride in pyridine (20 mg/mL). After vortexed for 1 min, the mixture was incubated at 37 °C for 90 min. Then 70 μL of MSTFA was added into the mixture and kept at 37 °C for 30 min after vortex-mixing. After at least 2 h at room temperature, the reaction mixture was analyzed by LC-ESI-MS/MS and GC–MS, respectively.

### Sample analysis

#### LC-ELSD analysis

LC-ELSD analysis was performed on an Agilent 1200 LC-Alltech 2000ES ELSD (Agilent, USA) equipped with a Prevail Carbohydrate ES pre-column (7.5 × 4.6 mm × 5 μm), and the targets were separated by a Prevail Carbohydrate ES chromatography column (250 × 4.6 mm × 5 μm) at 30 °C. The mobile phase (flow rate 1.0 mL/min) was a linear gradient prepared from water (A) and acetonitrile (B). The gradient program was (time, % A): 0–14 min, 15 %; 14–25 min, 15–35 %; 25–30 min, 35–45 %; 30–35 min, 45–15 %. The injection volume was 10 μL and the temperature for the flow shift tub in ELSD was 80 °C. The flow rate of N_2_ was 2.2 L/min with the striker of ELSD being closed.

#### LC-ESI-MS/MS analysis

The liquid chromatographic analysis was performed on a Waters Acquity UPLC instrument (Milford, MA, USA). Separation was carried out on an Acquity BEH C18 column (50 × 2.1 mm, 1.7 μm) maintained at 20 °C. The mobile phase consisted of solvent B and solvent C (10 mM ammonium formate in water). Initial gradient was set to 90 % B and held for 20 min, and then a linear gradient increasing to 95 % B until 30 min and maintained for 5 min. At 40 min the gradient was programmed to initial conditions to re-equilibrate the column for 5 min. The flow rate was 0.3 mL/min and the injection volume was 5 μL in full loop injection mode.

Detection was carried out by a Waters Xevo™ TQ triple-quadrupole MS fitted with ESI probe operated in the positive ion mode. The following parameters were optimal: capillary voltage, 3000 V; ion source temperature, 150 °C; desolvation gas temperature, 500 °C; desolvation gas flow rate, 800 L/h; collision gas, Argon; collision cell pressure, 4 mBar; multiple reactions monitoring (MRM) mode.

#### GC–MS analysis

Agilent 7890A gas chromatograph coupled to a 5975C mass spectrometer and a DB-5MS column (30 m length × 0.25 mm i.d. × 0.25 μm film thickness, J&W Scientific, USA) was employed for GC–MS analysis of sugars. Helium was used as carrier gas at a flow rate of 1 mL/min. The volume of injection was 1 μL and the split ratio was 10:1. The oven temperature was held at 70 °C for 4 min, and then raised to 310 at 5 °C/min and held at the temperature for 10 min. All samples were analyzed in both full scan (mass range of 40–510 amu) and selective ion scan mode. The injector inlet and transfer line temperature were 290 and 280 °C, respectively.

### Qualitative and quantitative analysis of sugars in jujube extract

Small molecular carbohydrates in jujube extract were identified by comparing retention time or mass fragment characteristic of targets with that of standard compounds, and NIST data and MS/MS were also employed for GC–MS and LC-ESI-MS/MS, respectively. Quantitative analysis was performed by calibration curve approach. All data presented in this paper are averages of five replicates unless otherwise stated. A mixed standard solution was prepared by dissolving the standard compound of rhamnose, xylitol, arabitol, fructose, glucose, inositol, sucrose, and maltose in unionized water, and diluted to a series of solution to obtain the calibration curves.

The standard solution with the lowest concentration of the calibration curves was analyzed for 10 times, and then their standard deviation (SD) was calculated. LOD and LOQ were defined, respectively, as three times of SD and ten times of SD [28]. The LOD value obtained using this method described here was comparable to those reported by Medeiros [29]. The sample J1 was employed to obtain the precision of the method, which was evaluated by relative standard deviation (RSD). Recovery experiment was performed on the spiked jujube extract at three spiking levels. The recoveries (five replicate tests) of analytes were calculated as (calculated amount/nominal amount) × 100 %.

## Results and discussion

### Method development and validation

#### HPLC-ELSD method

In order to measure small molecular carbohydrates by HPLC-ELSD, the jujube extract, a viscous liquid, was dissolved in unionized water and ultrosounded for 30 min. However, the supernatant was still turbid after centrifugation. Therefore, a purification step with solid phase extraction column was need for the analysis.

A series of experiments were carried out to select the SPE column. Fructose, glucose and sucrose used as targets were deposited into three different pre-treated SPE columns, including Bond Elut-C18, CNWBOND NH_2_ and Poly-Sery HLB, and eluted by water. The recoveries of the compounds were obtained to evaluate the performance of the SPE columns and summarized in Additional file 1: Table S1. The results indicated that Poly-Sery HLB column (mean recovery = 99–100.03 %, RSD = 0.1–0.8 %, n = 5) was more suitable in the purification of the sugars than Bond Elut-C18 (mean recovery = 90.31–94.77 %, RSD = 1.0–1.4 %, n = 5) and CNWBOND NH_2_ (mean recovery = 95.22–104.99 %, RSD = 1.0–2.2 %, n = 5). As a result, the SPE column was selected for our further experiment and the optimized conditions were that the sample volume and the eluting volume were both 1 mL.

Different type of LC chromatography column, such as Waters NH_2_ (250 × 4.6 mm), Waters Sugar-Pak I (300 × 6.5 mm), and Prevail Carbohydrate ES column were tried to separate the carbohydrates in jujube extraction. Prevail Carbohydrate ES column was selected to analyze the targets due to the Waters Sugar-Pak I column’s restriction in the mobile phase and the reaction of reducing sugar with NH_2_ group in Waters NH_2_ column. The optimized chromatographic conditions (the experimental data were showed as Additional file 1: Figure S1) were as follows: The mobile phase was a linear gradient prepared from A and B: (time, % A) 0–14 min, 15 %; 14–25 min, 15–35 %; 25–30 min, 35–45 %; 30–35 min, 45–15 %.

A series of mixed standard solutions were prepared in a concentration range of 10–2500 μg/mL, and six-point calibration curves of small molecular carbohydrates were constructed by the regression analysis of logarithm of chromatographic peek area of analyte (y) to concentration of analyte (x). The good linearity of response was achieved in an appropriate range with the coefficient of determination (R^2^ ≥ 0.9967). Limits of detection (LODs) and limits of quantitation (LOQs) were obtained in the range of 0.61–4.04 and 2.04–13.46 μg/mL, respectively. The data was summarized in Table 1.

**Table 1:** Analytical performance of the proposed method using LC-ELSD: linearity, LODs, and LOQs

Analyte	Calibration curves	Linear range (μg/mL)	*R* ^2^	LOD/ (μg/mL)	LOQ/ (μg/mL)
Rhamonse 鼠李糖	ln*y* = 2.2612ln*x* − 1.6630	50–1008	1.0000	4.04	13.46
Xylitol 木糖醇	ln*y* = 3.1110ln*x* − 3.6201	50–1003	0.9967	3.82	12.72
Arabitol 阿拉伯糖醇	ln*y* = 2.3304ln*x* + 0.9518	25–1010	0.9992	1.88	6.27
Fructose 果糖	ln*y* = 2.9108ln*x* − 2.3238	25–1000	0.9994	0.61	2.04
Glucose 葡萄糖	ln*y* = 1.9345ln*x* + 1.7266	50–1009	0.9998	1.03	3.44
Inositol 肌糖	ln*y* = 2.2319ln*x* + 2.1821	10–1008	0.9983	2.79	9.30
Sucrose 蔗糖	ln*y* = 2.2002ln*x* + 2.1501	10–1002	0.9992	2.39	7.98
Maltose 麦芽糖	ln*y* = 2.0900ln*x* + 1.7832	10–1009	0.9996	2.54	8.47

Repeatability and recovery were obtained to evaluate precision of the LC-ELSD method and the results were showed in Table 5. The repeatability, in terms of the relative standard deviation (RSD) of the replicate measurements, was judged to be satisfactory (RSD < 5.06 %, n = 5). The recovery of each analyte (showed as Additional file 1: Table S2) was obtained by the spiked jujube extract at three spiking levels and in the range of 94–105 %.

#### LC-ESI-MS/MS method

A step of derivatization is indispensable for small molecular carbohydrates to obtain a satisfactory analysis using LC-ESI-MS/MS. The derivatization step was carried out according to the method published in literatures [25–27]. Before derivatization, small molecular carbohydrates were oximated by reacting with methoxyamine hydrochloride to reduce the number of derivatives of reducing sugars [27]. Then the reaction mixture reacts directly with MSTFA to obtain the silylated product, which was analyzed by LC-ESI-MS/MS.

Working solutions of 1 mg/mL was infused to optimize the MS/MS parameters for each carbohydrate and internal standard. The ESI + mode was selected due to its sensitivity and easily handling and maintenance. A full scan mass spectrum was achieved to determine the precursor ions. And the most sensitive transitions were selected for quantification. The signal of each analyte was optimized by altering cone voltage (CV) and collision energies (CE). The selected transitions and the optimal MS/MS conditions are shown in Table 2.

**Table 2:** Retention time and LC-ESI-MS/MS parameters for analytes

Analyte	Retention time (min)	Derivative parent ion (m/z)	Derivative daughter ion (m/z)	Collision energy (V)	Cone voltage (V)
Xylose	3.86	468.30	217.20	22	16
Rhamnose	6.08	482.35	219.17	16	18
Xylitol	8.97	513.40	129.01	24	20
Glucose	10.66	570.40	307.28	18	14
Arabitol	11.35	513.40	129.10	26	20
Fructose	14.01	570.46	319.26	22	16
Inositol	33.02	613.40	191.20	26	28

However, the MS responses to sucrose and maltose were very poor even if the derivatization step was employed. The reason may be that the derivatives of disaccharides have larger molecular radius, therefore, the coulombic force cannot effectively overpower the surface tension, and the coulomb explosion affording charged microdroplets cannot occur successfully [29, 30]. So, sucrose and maltose were not identified by the LC-ESI-MS/MS method.

To obtain better resolution, different mobile phase systems were tried, including methanol–water, acetonitrile–water and acetonitrile–water adding ammonium formate or ammonium acetate. Under the starting condition of 90:10 B and C (V/V), the separation of the carbohydrates with satisfying peak shapes was achieved.

A series of working standard solutions of targets were prepared in the concentration range of 1–1000 μg/mL and analyzed by the LC-ESI-MS/MS method. Calibration curves were constructed by plotting the peak area ratio of analyte-to-internal standard (y) versus concentration of analyze (x). Table 3 showed that in all cases R^2^ values were beyond 0.9986, and the low LOD and LOQ observed revealed that the method had a satisfying sensitivity and it was suitable for the quantitative analysis.

**Table 3:** Analytical performance of the proposed method using LC-ESI-MS/MS: linearity, LODs, and LOQs

Analyte	Calibration curves	Linear range (μg/mL)	R^2^	LOD (μg/mL)	LOQ (μg/mL)
Rhamonse	y = 0.0029x − 0.0304	1.008–1008	0.9993	0.02	0.06
Xylitol	y = 0.0074x − 0.0712	1.008–1008	0.9993	0.06	0.19
Arabitol	y = 0.0107x − 0.0489	1.024–1024	0.9998	0.10	0.33
Fructose	y = 0.0138x + 0.1363	1.000–1000	0.9986	0.13	0.42
Glucose	y = 0.0058x − 0.0264	1.032–1032	0.9999	0.01	0.04
Inositol	y = 0.0215x + 0.0077	1.012–1012	0.9989	0.01	0.03

Precision of the LC-ESI-MS/MS method was evaluated and the results were deposited in Table 5. The RSD was less than 5 % for five replicate measurements. The recoveries produced at three spiking levels were in the range of 87–110 % among the individual analytes (showed as Additional file 1: Table S3).

#### GC–MS method

Similar to LC-ESI-MS/MS, GC–MS method also required a derivatization step to achieve the analysis of small molecular carbohydrates. In this study, the GC–MS method reported in the literature [27], with a little change, was employed to perform on the analysis of small molecular carbohydrates in jujube extract. The derivatization step, which was identical with that of the LC-ESI-MS/MS method, was included in the GC–MS method. The silylated products of analytes were analyzed by GC–MS.

The linearity study of the method was made by preparing seven mixed working standard solutions covering the concentration range of 1–1000 μg/mL, derivatizing, and analyzing by GC–MS. The calibration curves and performance characteristics of the method were summarized in Table 4, and the results showed that calibration curve for each analyte had a good linear regression (R^2^ = 0.9946–0.9998) in the range.

**Table 4:** Analytical performance of the proposed method using GC–MS: linearity, LODs, and LOQs

Analyte	Calibration curves	Linear range (μg/mL)	R^2^	LOD (μg/mL)	LOQ (μg/mL)
Rhamonse	y = 0.0005x − 0.0096	1.040–1040	0.9946	0.88	2.95
Xylitol	y = 0.0008x − 0.0088	1.032–1032	0.9978	0.53	1.76
Arabitol	y = 0.0010x − 0.0070	1.024–1024	0.9995	0.49	1.62
Fructose	y = 0.0003x − 0.0057	1.032–1032	0.9959	0.17	0.56
Glucose	y = 0.0009x − 0.0099	1.020–1020	0.9953	0.65	2.15
Inositol	y = 0.0013x + 0.003	1.024–1024	0.9998	0.20	0.68
Sucrose	y = 0.0005x − 0.0103	1.020–1020	0.9948	0.29	0.95
Maltose	y = 9E−05x − 0.0005	1.020–1020	0.9978	0.58	1.93

Repeatability and recovery studies were carried out to evaluate precision and accuracy of the GC–MS method, and the results were summarized in Table 5. The coefficients of variation of analytes were fine. The recoveries were in the range 68–109 % among the individual sugars (showed as Additional file 1: Table S4).

**Table 5:** Comparisons of precision and accuracy from the methods with HPLC-ELSD, LC-ESI-MS/MS, and GC–MS

Analytes	HPLC-ELSD					LC-ESI-MS/MS					GC–MS				
	Mean, mg/g		LOD μg/mL	RSD %	MR %	Mean, mg/g		LOD μg/mL	RSD %	MR %	Mean, mg/g		LOD μg/mL	RSD %	MR %
	J1	J2				J1	J2				J1	J2			
Rhamnose	ND	ND	4.04	–	98.33	3.91	3.26	0.02	3.61	101.73	4.09	3.59	0.88	11. 05	86.47
Xylitol	ND	ND	3.82	–	100.33	3.41	2.81	0.06	3.28	87.47	3.52	2.88	0.53	10.77	68.73
Arabitol	ND	ND	1.88	–	94.00	1.66	1.42	0.10	4.53	88.60	1.69	1.43	0.49	12.58	76.03
Fructose	165.25	143.66	0.61	2.05	105.00	164.040	143.78	0.13	3.72	99.00	166.84	144.14	0.17	8.42	102.20
Glucose	189.89	177.21	1.03	4.00	104.67	187.14	180.44	0.01	4.97	99.50	190.11	181.33	0.65	7.19	104.37
Inositol	4.15	3.15	2.79	5.06	95.33	4.04	2.99	0.01	2.33	109.35	4.52	3.13	0.20	10.93	80.00
Sucrose	18.04	20.18	2.39	1.27	102.33	–	–	–	–	–	17.65	19.79	0.29	8.64	108.47
Maltose	7.15	5.13	2.54	3.91	97.67	–	–	–	–	–	6.97	4.91	0.58	9.31	103.73

*MR* mean recovery, calculated as a mean of low, middle, high spiked recovery; *ND* no detected; – unable to detect

### Comparison of HPLC-ELSD, LC-ESI-MS/MS and GC–MS for small molecular carbohydrates in jujube extract

Jujube extract dissolved in unionized water were used to compare the feasibility of the methods for small molecular carbohydrates. All the eight small molecular carbohydrates were determined by the validated methods, including rhamnose, xylitol, arabitol, fructose, glucose, inositol, sucrose and maltose. Typical chromatograms of small molecular carbohydrates in jujube extract by the three methods were shown in Additional file 1: Figures S2, S3 and S4. The results were summarized in Table 5.

The GC–MS method can detect all eight sugars and alditols in jujube extract, whereas that is five for the HPLC-ELSD method and six for the LC-ESI-MS/MS method. Although only three sugars including fructose, glucose and inositol can be determined from jujube extract by all these methods, the data obtained were very similar. The results indicated that all the three the methods could be used to determine sugars in jujube extract under appropriate conditions, however, the methods were different in their applicability.

The data, deposited in Tables 1 and 5, indicated that sample with a simple pretreating can be directly analyzed by HPLC-ELSD to achieve good linearity, recovery, repeatability, and acceptable sensitivity, which makes the method the optimal choice for a routine analysis of sugars and alditols in jujube extract. Despite all of this, the value of LODs by the HPLC-ELSD method is beyond 1 μg/mL except fructose in this study, and some compounds present in jujube extract with low level were not detected by the HPLC-ELSD method, such as rhamnose, xylitol and arabitol. Smaller values of LODs were also reported in the analysis of sugars in fruits [31]. One reason was that the value of LODs in the reference was on the basis of response and slope of each regression equation at a signal-to-noise ratio(S/N) of 3, other reason may be that the SPE purified step was added into our method. In addition, the HPLC-ELSD method has no ability to provide the structure information of analytes due to the ELSD detector. As a result, standard substance was indispensable for identifying the targets, which restricted its application in qualitative analysis.

The data, deposited in Tables 3, 4, showed that good linearity in a wider concentration range can be obtained by the LC-ESI-MS/MS method and the GC–MS method, respectively. Compared with the HPLC-ELSD method, both of the two MS methods can achieve better sensitivity. Most analytes can be detected reliably by LC-ESI-MS/MS (LOD < 0.1 μg/mL) and GC–MS (LOD < 0.5 μg/mL) at low concentration (initial reactants), respectively. However, a derivatization step was indispensable for LC-ESI-MS/MS and GC–MS to achieve the analysis of sugars and alditols, and the derivatization products were very complex because reducing sugars usually have varieties of isomers that co-exist in aqueous solution, which would increase the difficulty for qualitative and quantitative analysis by chromatographic technique. Although the derivatization method involving oximation can successfully reduce the number of derivatization products, the two step procedure costs the simplicity, recovery and repeatability of the MS methods.

Table 5 showed that both the recoveries of GC–MS method and LC-ESI-MS/MS method were inferior to those of HPLC-ELSD method in spite of the fact that all of them were in an acceptable range. Obviously, the poor repeatability (RSD > 7 %) was a drawbacks of the GC–MS method to analyze sugars and alditols in jujube extract. And the lower concentrations of analytes, the worse repeatability obtained by GC–MS method. The reasons might be that the complex sample pretreating process results in loss of sample and instability of the derivatization products in high moisture environment [32]. But, the MS methods have better sensitivity and can provide chemical structure information of analytes, which made them remarkably advantageous in qualitative analysis, especially for trace component in sample matrix.

Following the data, the performance of the LC-ESI-MS/MS method preceded the GC–MS method in the mass when they were applied to analysis of sugars and alditols in jujube extract. Some possible reasons included the higher injection volume used in LC-ESI-MS/MS (5 vs. 1 μL), the lower amount of fragmentation during ionization (ESI vs. EI) [14], etc. The data also indicated that lower value of carbohydrates content was obtained using the LC-ESI-MS/MS method, which could be due to the reduced matrix interference as tandem MS was used. Different molecules that share the same transition are more rarely found than molecules producing fragments of identical mass [14], as a result, peak identification and integration are much easier by LC-ESI-MS/MS, and require less manual corrections [15]. So, better repeatability can also be obtained by the LC-ESI-MS/MS method, which made the method more suitable than the GC–MS method for quantitative analysis of sugars and alditols in jujube extract. The LC-ESI-MS/MS method was ineffective for disaccharides, however, other methods would be needed to determine sucrose and maltose in jujube extract.

## Conclusions

HPLC-ELSD, LC-ESI-MS/MS and GC–MS methods were respectively developed and applied to the analysis of small molecular carbohydrates in jujube extract. All the eight sugars and alditols were determined, including rhamnose, xylitol, arabitol, fructose, glucose, inositol, sucrose, and maltose. Although the methods have been found to perform satisfactorily, only three sugars could be detected by all these methods. The results indicated that a multi-analysis technique should be adopted in order to obtain complete qualitative and quantitative information of small molecular carbohydrates in jujube extract.

The performance characteristics of the three methods were compared by precision and accuracy, which showed that the HPLC-ELSD method with a simple pretreating step can achieve good repeatability, recovery and acceptable sensitivity and was very suitable for quantitative analysis; whereas the MS methods were more sensitive and provided chemical structure information of targets, therefore, more suitable for qualitative analysis. The benefits of LC-ESI-MS/MS in terms of higher sensitivity, better repeatability and higher selectivity are obvious, so it was also suitable for quantitative analysis. Although the performance of the GC–MS method for quantitative analysis was inferior to the other methods, it had a wider scope on identification of small molecular carbohydrates and was suitable for qualitative analysis. So, the methods should be employed according to the purpose of analysis.

## Additional file

10.1186/s13065-016-0171-2 **Table S1** The recoveries of carbohydrates in Jujube extract with different SPE cartridges. **Table S2** Recoveries of eight carbohydrates in sample by the HPLC-ELSDmethod (n = 5). **Table S3** Recoveries of six carbohydrates in sample by the LC-ESI-MS/MS method (n = 5). **Table S4** Recoveries of eight carbohydrates in sample by the GC-MS method (n = 5). **Figure S1** The comparison among separation performances of nine analytes under three different elution modes. The mobile phase (flow rate 1.0 mL/min) was a linear gradient prepared from water (A) and acetonitrile (B). a. isocratic elution: 20 % A + 80 % B, (v/v); b. gradient elution: 15 % A (Initial gradient), then increasing to 30 % A until 30 min and held for 5 min; c. The gradient program was (time, % A): 0–14 min, 15 %; 14–25 min, 15–35 %; 25–30 min, 35–45 %; 30-35 min, 45–15 %; *1* rhamnose, *2* xylitol, *3* arabitol, *4* fructose, *5* glucose, *6* inositol, *7* sucrose, *8* maltose. **Figure S2** Representative HPLC-ELSD chromatogram of small molecular carbohydrates in jujube extract: *1* rhamnose, *2* xylitol, *3* arabitol, *4* fructose, *5* glucose, *6* inositol, *7* sucrose, *8* maltose. a. Standard substances; b. Jujube extract. **Figure S3** (A) The MRM chromatograms of xylose (internal standard), rhamnose, xylitol, glucose, arabitol, fructose and inositol in standard solution. (B) The MRM chromatograms of jujube extract sample. **Figure S4** Representative GC-MS chromatogram of small molecular carbohydrates in jujube extract: *1* xylose (internal standard), *2* xylitol, *3* rhamnose, *4* arabitol, *5* fructose, *6* glucose, *7* inositol, *8* surcrose, *9* maltose. (A) Standard substances. (B) Jujube extract.
