# Supplementary material for: Simultaneous determination of rhamnose, xylitol, arabitol, fructose, glucose, inositol, sucrose, maltose in jujube (Zizyphus jujube Mill.) extract: comparison of HPLC–ELSD, LC–ESI–MS/MS and GC–MS
Source: Chem Cent J. 2016 Apr 30;10:25. doi: 10.1186/s13065-016-0171-2 (PMC4852422; doi:10.1186/s13065-016-0171-2)
Supplement: Supplementary file 1 — 10.1186/s13065-016-0171-2 Table S1 The recoveries of carbohydrates in Jujube extract with different SPE cartridges. Table S2 Recoveries of eight carbohydrates in sample by the HPLC-ELSDmethod (n = 5). Table S3 Recoveries of six carbohydrates in sample by the LC-ESI-MS/MS method (n = 5). Table S4 Recoveries of eight carbohydrates in sample by the GC-MS method (n = 5). Figure S1 The comparison among separation performances of nine analytes under three different elution modes. The mobile phase (flow rate 1.0 mL/min) was a linear gradient prepared from water (A) and acetonitrile (B). a. isocratic elution: 20 % A + 80 % B, (v/v); b. gradient elution: 15 % A (Initial gradient), then increasing to 30 % A until 30 min and held for 5 min; c. The gradient program was (time, % A): 0–14 min, 15 %; 14–25 min, 15–35 %; 25–30 min, 35–45 %; 30-35 min, 45–15 %; 1 rhamnose, 2 xylitol, 3 arabitol, 4 fructose, 5 glucose, 6 inositol, 7 sucrose, 8 maltose. Figure S2 Representative HPLC-ELSD chromatogram of small molecular carbohydrates in jujube extract: 1 rhamnose, 2 xylitol, 3 arabitol, 4 fructose, 5 glucose, 6 inositol, 7 sucrose, 8 maltose. a. Standard substances; b. Jujube extract. Figure S3 (A) The MRM chromatograms of xylose (internal standard), rhamnose, xylitol, glucose, arabitol, fructose and inositol in standard solution. (B) The MRM chromatograms of jujube extract sample. Figure S4 Representative GC-MS chromatogram of small molecular carbohydrates in jujube extract: 1 xylose (internal standard), 2 xylitol, 3 rhamnose, 4 arabitol, 5 fructose, 6 glucose, 7 inositol, 8 surcrose, 9 maltose. (A) Standard substances. (B) Jujube extract. [file 13065_2016_171_MOESM1_ESM.docx]

**Table S1 The recoveries of carbohydrates in Jujube extract with different SPE cartridges**

| Type of  SPE columns | | Bond Elut-C18 | Bond Elut-C18 | CNWBOND NH_2_ | CNWBOND NH_2_ | Poly-Sery HLB | Poly-Sery HLB |
| --- | --- | --- | --- | --- | --- | --- | --- |
| Eluting volume, mL | | 1.0 | 2.0 | 1.0 | 2.0 | 1.0 | 2.0 |
| Fructose, % | 1 | 87.71 | 93.11 | 83.14 | 96.22 | 97.92 | 98.55 |
|  | 2 | 91.92 | 94.72 | 89.02 | 95.15 | 99.71 | 93.12 |
|  | 3 | 90.00 | 95.19 | 88.15 | 96.33 | 99.35 | 97.91 |
|  | 4 | 88.32 | 94.08 | 84.01 | 92.07 | 98.67 | 93.00 |
|  | 5 | 92.05 | 93.03 | 87.72 | 96.32 | 99.62 | 95.78 |
|  | Mean | 90.00 | 94.03 | 86.41 | 95.22 | 99.05 | 95.67 |
| RSD, % | | 2.2 | 1.0 | 3.1 | 1.9 | 0.8 | 2.7 |
| Glucose, % | 1 | 82.50 | 89.09 | 73.05 | 98.31 | 99.97 | 109.50 |
|  | 2 | 87.44 | 92.21 | 80.63 | 97.47 | 100.03 | 107.95 |
|  | 3 | 87.01 | 91.11 | 78.77 | 95.99 | 99.94 | 113.27 |
|  | 4 | 85.07 | 91.02 | 77.38 | 97.10 | 100.17 | 115.66 |
|  | 5 | 85.00 | 90.31 | 78.65 | 96.08 | 99.81 | 109.14 |
|  | Mean | 85.40 | 90.75 | 77.70 | 96.99 | 99.98 | 111.10 |
| RSD, % | | 2.3 | 1.3 | 3.7 | 1.0 | 0.1 | 2.9 |
| Sucrose, % | 1 | 82.18 | 95.07 | 91.00 | 101.29 | 99.83 | 101.88 |
|  | 2 | 84.79 | 96.48 | 92.11 | 104.64 | 100.04 | 105.37 |
|  | 3 | 85.01 | 95.23 | 93.70 | 107.49 | 100.25 | 100.00 |
|  | 4 | 85.26 | 94.05 | 87.06 | 106.00 | 100.60 | 97.24 |
|  | 5 | 84.73 | 93.04 | 89.86 | 105.51 | 99.41 | 96.24 |
|  | Mean | 84.39 | 94.77 | 90.75 | 104.99 | 100.03 | 100.15 |
| RSD, % | | 1.5 | 1.4 | 2.8 | 2.2 | 0.4 | 3.7 |

**Table S2 Recoveries of eight carbohydrates in sample by the HPLC-ELSDmethod (*n*=5)**

| Targets | Nominal amount  (mg/g) | Calculated amount  (mg/g) | Mean Recovery  (%) | RSD  (%) |
| --- | --- | --- | --- | --- |
| Rhamonse | 4.99 | 4.74 | 94.9 | 2.35 |
|  | 10.21 | 9.90 | 97.0 | 7.31 |
|  | 20.04 | 20.64 | 103.0 | 9.77 |
| Xylitol | 4.89 | 5.09 | 104.1 | 6.78 |
|  | 10.07 | 10.37 | 103.0 | 7.43 |
|  | 20.13 | 18.92 | 94.0 | 5.22 |
| Arabitol | 5.11 | 5.06 | 99.0 | 4.65 |
|  | 10.08 | 9.17 | 91.0 | 5.07 |
|  | 20.19 | 18.57 | 92.0 | 7.77 |
| Fructose | 101.29 | 108.38 | 107.0 | 1.23 |
|  | 184.16 | 195.02 | 105.9 | 1.42 |
|  | 358.77 | 366.54 | 102.2 | 7.11 |
| Glucose | 83.42 | 89.26 | 107.0 | 0.74 |
|  | 174.26 | 183.24 | 105.2 | 1.76 |
|  | 338.95 | 346.62 | 102.3 | 8.50 |
| Inositol | 2.50 | 2.25 | 90.0 | 4.88 |
|  | 4.95 | 5.20 | 105.1 | 1.15 |
|  | 11.89 | 10.82 | 91.0 | 3.05 |
| Sucrose | 10.92 | 11.39 | 104.3 | 1.72 |
|  | 17.82 | 18.75 | 105.2 | 3.56 |
|  | 38.65 | 37.97 | 98.2 | 9.60 |
| Maltose | 4.97 | 5.11 | 102.8 | 4.03 |
|  | 7.92 | 7.69 | 97.1 | 4.27 |
|  | 17.84 | 16.53 | 92.6 | 7.80 |

**Table S3** **Recoveries of six carbohydrates in sample by the LC-ESI-MS/MS method (*n*=5)**

| Targets | Nominal amount  (mg/g) | Calculated amount  (mg/g) | Mean Recovery  (%) | RSD  (%) |
| --- | --- | --- | --- | --- |
| Rhamonse | 2.01 | 2.14 | 106.6 | 5.90 |
|  | 4.03 | 3.97 | 98.5 | 3.96 |
|  | 8.24 | 9.07 | 100.1 | 7.19 |
| Xylitol | 1.72 | 1.49 | 86.6 | 9.43 |
|  | 3.51 | 3.22 | 91.7 | 6.01 |
|  | 7.00 | 5.89 | 84.1 | 7.92 |
| Arabitol | 0.90 | 0.82 | 91.1 | 6.93 |
|  | 1.70 | 1.41 | 82.9 | 5.79 |
|  | 3.53 | 3.21 | 91.0 | 8.44 |
| Fructose | 81.13 | 81.14 | 100.0 | 6.37 |
|  | 165.20 | 166.69 | 100.9 | 4.81 |
|  | 330.40 | 317.5 | 96.1 | 6.92 |
| Glucose | 95.50 | 97.2 | 101.8 | 7.03 |
|  | 190.86 | 189.12 | 99.1 | 8.24 |
|  | 381.00 | 371.86 | 97.6 | 6.11 |
| Inositol | 2.31 | 2.66 | 115.2 | 8.97 |
|  | 4.27 | 4.41 | 103.3 | 5.15 |
|  | 8.57 | 9.51 | 111.0 | 6.04 |

**Table S4 Recoveries of eight carbohydrates in sample by the GC-MS method (*n*=5)**

| Targets | Nominal amount  (mg/g) | Calculated amount  (mg/g) | Mean recovery  (%) | RSD  (%) |
| --- | --- | --- | --- | --- |
| Rhamonse | 2.01 | 1.79 | 89.1 | 11.02 |
|  | 4.03 | 3.61 | 89.6 | 10.27 |
|  | 8.24 | 6.65 | 80.7 | 9.16 |
| Xylitol | 1.72 | 1.22 | 70.9 | 13.29 |
|  | 3.51 | 2.22 | 63.2 | 12.72 |
|  | 7.00 | 5.05 | 72.1 | 8.50 |
| Arabitol | 0.90 | 0.56 | 62.2 | 15.67 |
|  | 1.70 | 1.33 | 78.2 | 12.44 |
|  | 3.53 | 3.08 | 87.7 | 10.87 |
| Fructose | 81.13 | 85.67 | 105.6 | 7.70 |
|  | 165.20 | 168.34 | 101.9 | 8.11 |
|  | 330.40 | 327.43 | 99.1 | 8.32 |
| Glucose | 95.50 | 103.62 | 108.5 | 6.55 |
|  | 190.86 | 199.83 | 104.7 | 9.74 |
|  | 381.00 | 380.62 | 99.9 | 8.46 |
| Inositol | 2.31 | 1.77 | 76.6 | 11.19 |
|  | 4.27 | 3.56 | 83.4 | 10.66 |
|  | 8.57 | 6.86 | 80.0 | 10.21 |
| Sucrose | 9.12 | 9.38 | 102.9 | 8.56 |
|  | 17.25 | 18.87 | 109.4 | 9.36 |
|  | 34.50 | 39.02 | 113.1 | 9.61 |
| Maltose | 3.51 | 3.86 | 110.1 | 13.38 |
|  | 6.98 | 7.20 | 103.2 | 12.77 |
|  | 13.56 | 13.28 | 97.9 | 8.93 |


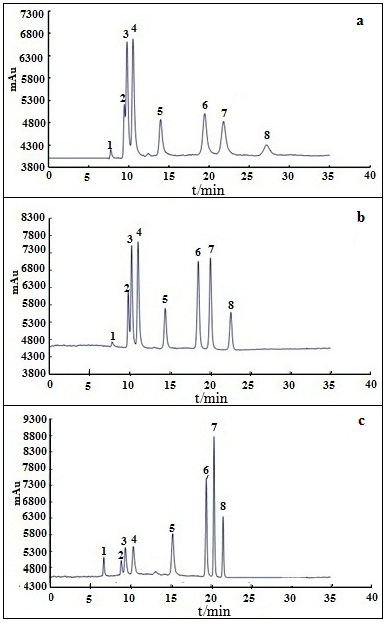


**Figure S1** The comparison among separation performances of nine analytes under three different elution modes. The mobile phase (flow rate 1.0 mL/min) was a linear gradient prepared from water (A) and acetonitrile (B). a. isocratic elution: 20% A + 80% B, (v/v); b. gradient elution:15% A (Initial gradient)，then increasing to 30% A until 30 min and held for 5 min; c. The gradient program was (time, % A): 0–14 min, 15%; 14-25 min, 15%-35%; 25-30 min, 35%-45%; 30-35 min, 45%-15%; 1. rhamnose; 2.xylitol; 3. arabitol; 4. fructose; 5. glucose; 6. inositol; 7. sucrose; 8) maltose.


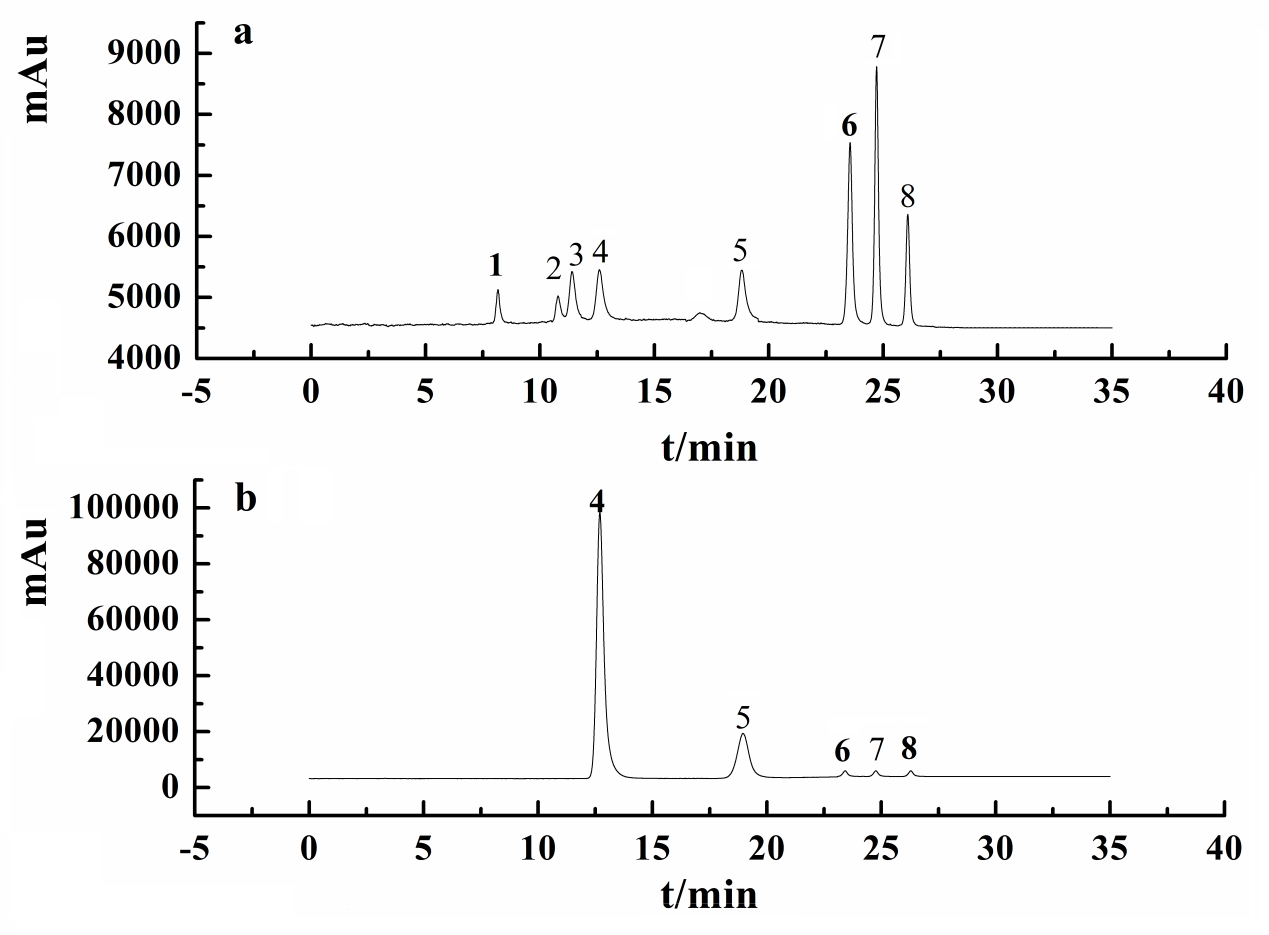


**Figure** **S2** Representative HPLC-ELSD chromatogram of small molecular carbohydrates in jujube extract: 1) rhamnose; 2) xylitol; 3) arabitol; 4) fructose; 5) glucose; 6) inositol; 7) sucrose; 8) maltose. a. Standard substances; b. Jujube extract.


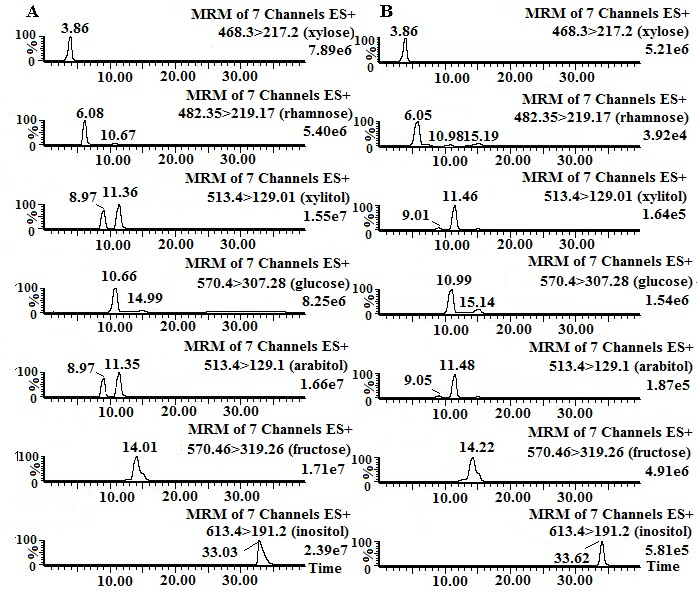


**Figure S3** (A) The MRM chromatograms of xylose (internal standard), rhamnose, xylitol, glucose, arabitol, fructose and inositol in standard solution. (B) The MRM chromatograms of jujube extract sample.


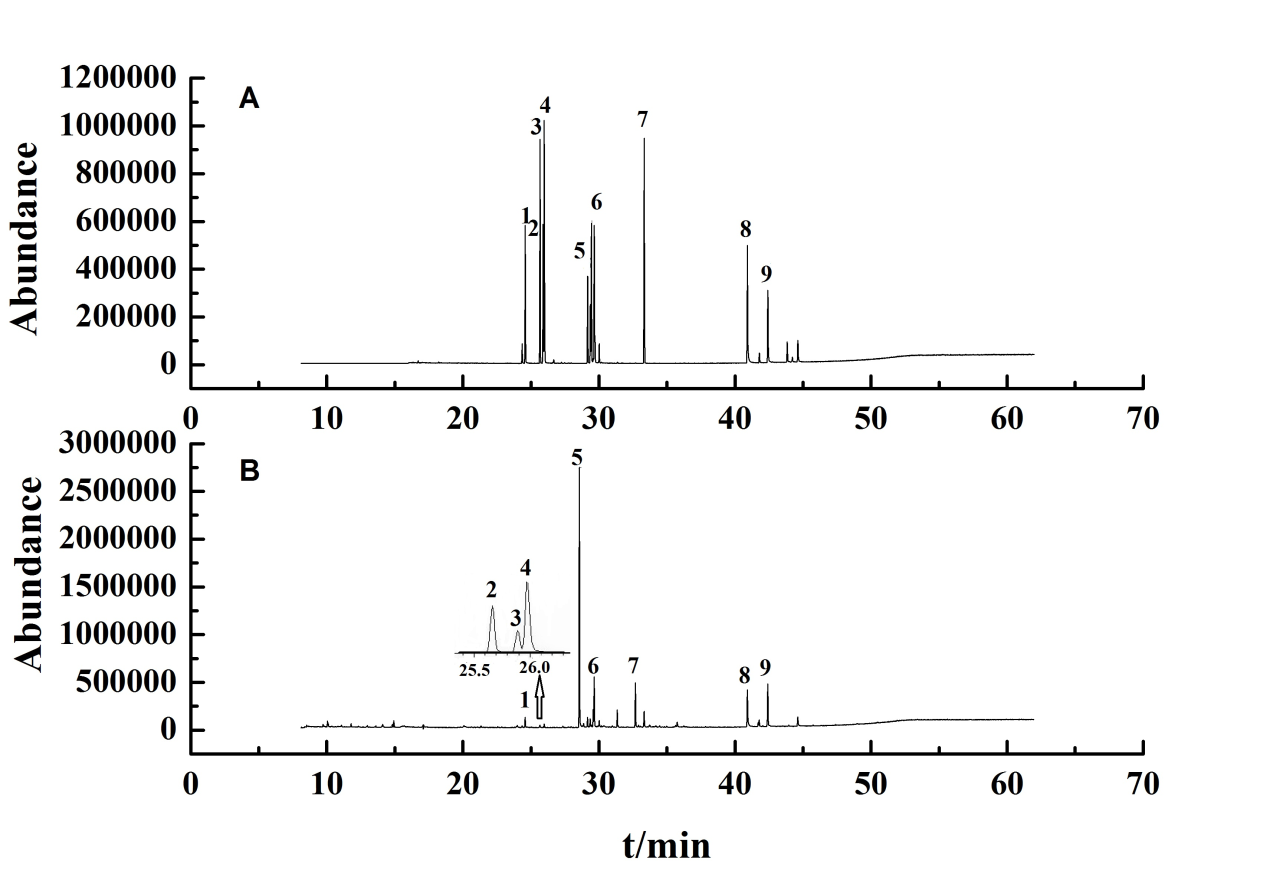


**Figure S4** Representative GC-MS chromatogram of small molecular carbohydrates in jujube extract: 1) xylose (internal standard); 2) xylitol; 3) rhamnose; 4) arabitol ; 5) fructose; 6) glucose; 7) inositol; 8) surcrose; 9) maltose.(A) Standard substances. (B) Jujube extract.
